# PREDICTOR: A Physical emulatoR enabling safEty anD ergonomICs evaluation and Training of physical human-rObot collaboRation

**DOI:** 10.3389/fnbot.2023.1080038

**Published:** 2023-02-13

**Authors:** Carl Emil Sunesson, Daniel Tofte Schøn, Christopher Nybo Ploug Hassø, Francesco Chinello, Cheng Fang

**Affiliations:** ^1^SDU Robotics, The Maersk Mc-Kinney Moller Institute, University of Southern Denmark, Odense, Denmark; ^2^Business Development and Technology, Aarhus University, Herning, Denmark

**Keywords:** physical human-robot collaboration, physical emulator, safety and ergonomics evaluation, training, haptic interface

## Abstract

Safety and ergonomics of Physical Human-Robot Collaboration (PHRC) are crucial to make human-robot collaborative systems trustworthy and make a significant impact in real-world applications. One big obstacle to the development of relevant research is the lack of a general platform for evaluating the safety and ergonomics of proposed PHRC systems. This paper aims to create a Physical emulatoR enabling safEty anD ergonomICs evaluation and Training of physical human-rObot collaboRation (PREDICTOR). PREDICTOR consists of a dual-arm robot system and a VR headset as its hardware and contains physical simulation, haptic rendering and visual rendering modules as its software. The dual-arm robot system is used as an integrated admittance-type haptic device, which senses the force/torque applied by a human operator as an input to drive the simulation of a PHRC system and constrains the handles' motion to match their virtual counterparts in the simulation. The motion of the PHRC system in the simulation is fed back to the operator through the VR headset. PREDICTOR combines haptics and VR to emulate PHRC tasks in a safe environment since the interactive forces are monitored to avoid any risky events. PREDICTOR also brings flexibility as different PHRC tasks can be easily set up by changing the PHRC system model and the robot controller in the simulation. The effectiveness and performance of PREDICTOR were evaluated by experiments.

## 1. Introduction

Undoubtedly, robots are becoming intimate and ubiquitous in our lives. Interactive robotics research and innovation is a megatrend in the new era of robotics. Collaborative robots (cobots), which are supposed to be deployed and work in the proximity of humans, have already demonstrated high potential in manufacturing systems (Djuric et al., 2016), construction sites (Liu et al., 2021) and even human-inhabited environments such as hospitals (Vogel et al., 2020b) and age- or disabled-appropriate houses (Vogel et al., 2020a). It is well recognized that cobots are indispensable elements in future factories as they can bring high flexibility and adaptability to the industrial processes for flexible and agile manufacturing as a featured trend in Industry 4.0 (Shi et al., 2012), and they can also contribute to improving the quality of people's daily life (Veloso et al., 2015).

Physical Human-Robot Collaboration (PHRC) is a special type of physical human-robot interaction, and it is defined when human(s), cobot(s) and the environment come to intentional and continuous contact(s) with each other and form a coupled dynamic system to accomplish a task (Bauer et al., 2008; Krüger et al., 2009). Future generation of cobots are envisioned to get closer to humans and can realize PHRC tasks in more challenging applications by tightly combining cobot physical capability and human cognitive ability. For instance, when a human worker and a cobot co-manipulate a car engine and transport it to a target place, the cobot can take most of the payload while the human worker can lead the engine to the target place and perform fine adjustment of the engine's position and orientation for further accurate operations, e.g., assembly. This is a typical scenario where PHRC is necessarily required. Similar scenarios can be easily found wherever fine manipulation of bulky and/or heavy objects is required, e.g., in healthcare (co-transporting patients), construction (co-assembly), logistics (co-transportation) domains, among others.

Research in PHRC has been active and popular in academia, and relevant progress and advances have been made in many aspects including control schemes, interaction modalities and interfaces for improved human and robot perception (Ajoudani et al., 2018b). To turn these research investigations into trustworthy technologies, which can make impact in real-world applications, safety is of paramount importance for the deployment of PHRC systems (Gualtieri et al., 2021). A large body of research work has been conducted on safety issues concerning unintentional collisions between human and cobot. Seven sequential phases of a collision event have been recognized and studied in literature including Pre-Collision (Khatib, 1985), Collision Detection (Suita et al., 1995), Collision Isolation (Yamada et al., 1997), Collision Identification (De Luca and Mattone, 2005), Collision Classification (Golz et al., 2015), Collision Reaction (Haddadin et al., 2008) and Post-Collision (Parusel et al., 2011), and they have been systematically summarized within a single safety framework in Haddadin et al. (2017), and some of them have been implemented as an open-source library, OpenPHRI (Navarro et al., 2017). This framework is, however, a collision-based safety framework in physical human-robot interaction where the goal is to avoid unintentional contacts/collisions. In contrast, the role of contact is rethought in PHRC, and intentional contacts are actively utilized to connect the human and the robot to form a powerful collaborative system and achieve cohesive synergy. But safety study of PHRC has been rarely touched and a PHRC safety framework is completely missing (Bi et al., 2021). Consequently, PHRC has been discussed extensively in academia, but rarely seen in industries. A key reason for the lack of systematic safety research is real PHRC experiments (usually many trials) regarding safety evaluation are typically dangerous, cumbersome and inconvenient.

On the other hand, ergonomics research in PHRC is recently emerging (Gualtieri et al., 2021). Ergonomics in PHRC is concerned with chronic health risks or illness incurred by inappropriate repetitive interactions between humans and cobots in static or quasi-static scenarios (e.g., human-robot co-assembly and co-drilling) while safety in PHRC is associated with instant physical injuries caused by more dynamic interactions (e.g., human-robot co-lifting and co-transportation). A collaborative cell was developed to improve the operator ergonomics during human-robot co-assembly tasks (Cherubini et al., 2016). A fatigue management framework in PHRC tasks was proposed by utilizing the human muscle force estimation based on a musculoskeletal model (Peternel et al., 2018). A reconfigurable human-robot collaboration workstation was developed to improve the worker ergonomics and productivity during co-manipulation tasks (Kim et al., 2019). A digital human model was used to simulate PHRC tasks for improving the design of cobots in terms of human ergonomics in Maurice et al. (2017) where a co-drilling task was demonstrated for the process of cobot design optimization. The ergonomics assessment and intervention are usually conducted either on real systems in real time, where preparing and setting up the whole system is time-consuming, or in pure simulations where the accuracy of the human model and its reactive behavior are questionable.

A big obstacle to the development of safety and ergonomics research in PHRC is the lack of a general platform for assessing the safety and ergonomics of PHRC tasks. This paper is targeted at this issue, and the contribution lies in the creation of a PHRC physical emulator, PREDICTOR, as a new tool enabling the safety and ergonomics evaluation of PHRC. PREDICTOR combines haptics and Virtual Reality (VR) to enable physical interaction between real human operator and virtual cobot through virtual co-manipulated object in PHRC. Real human operator is involved instead of digital human model for real reactive behavior and dangerous heavy/bulky object and robot are simulated for safety reason, which are the main benefits of PREDICTOR. It can predict what will happen in a real counterpart in order to optimize the control schemes and/or tasks, and train human operators before real PHRC experiments.

## 2. Overview of PREDICTOR

### 2.1. System hardware

**Figure 1:**
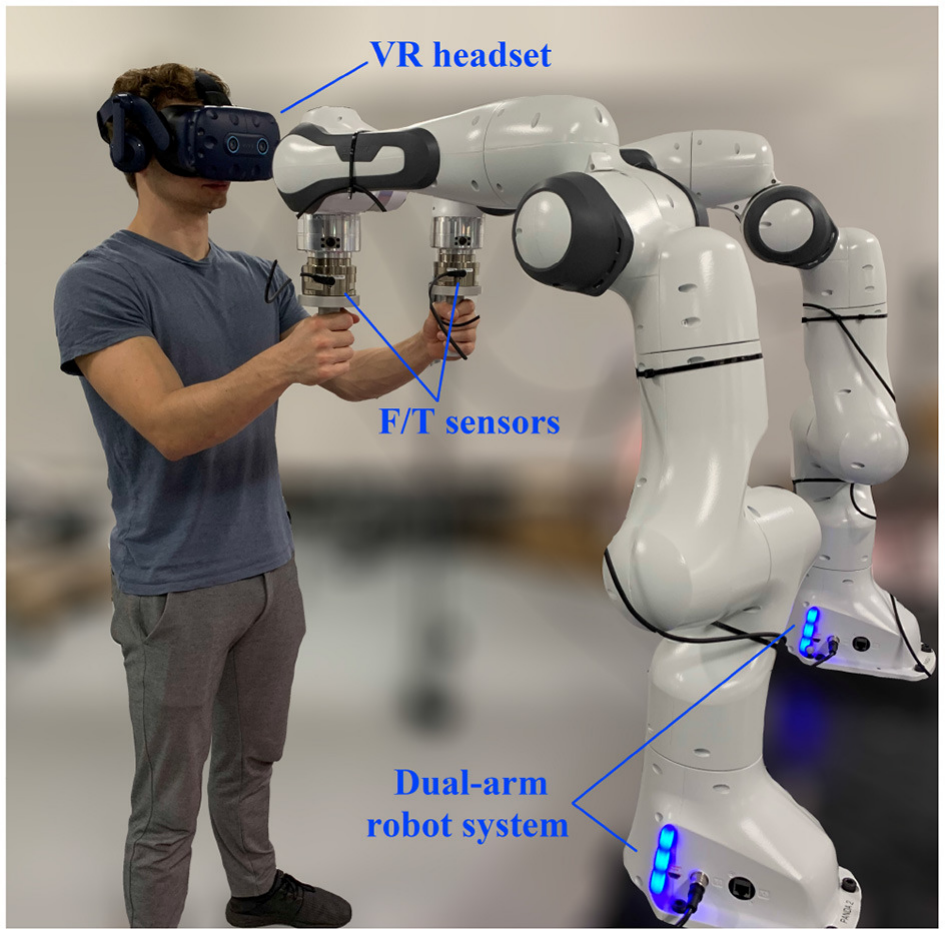
Setup of PREDICTOR consisting of a dual-arm robot system (Franka Emika Panda) and a VR headset (HTC VIVE Pro Eye).

As shown in Figure 1, PREDICTOR consists of a single- or dual-arm cobot system and a VR headset. A human operator uses one or two hand(s) to grip one or two real handle(s) mounted at the end-effector(s) of cobot(s) (depending on the task type). The cobot system is used as an integrated haptic device, which makes the operator feel as if he/she co-manipulated a virtual object with a virtual cobot, which can be seen through a VR headset. Virtual handle(s) corresponding to the real handle(s) is/are attached to the virtual object. It is worth mentioning that the PREDICTOR setup might resemble some teleoperation (Hulin et al., 2011) or telepresence (Buss et al., 2010) systems at first glance, but they function completely differently: while the operator uses two master arms to teleoperate two real remote arms independently in the teleoperation and telepresence, the operator uses the two master arms/real handles as part of a single virtual object co-manipulated by a virtual robot in the physical emulation of PHRC.

### 2.2. Software modules

**Figure 2:**
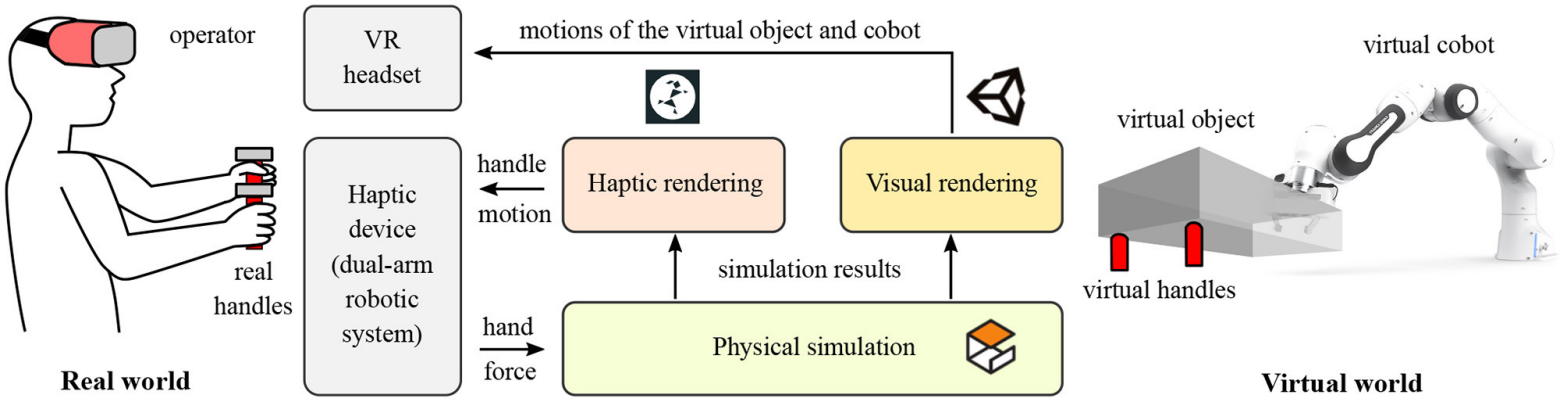
Schematic diagram of PREDICTOR and its components. The hardware includes a dual-arm robotic system as a haptic device and a VR headset. The software consists of three modules including physical simulation, haptic rendering and visual rendering modules.

As shown in Figure 2, The software of PREDICTOR includes three modules: physical simulation, haptic rendering and visual rendering modules. The physical simulation module is responsible for the physical simulation of the dynamic behavior of the coupled human-robot collaborative system, which is implemented in an open-source robotics simulator, Gazebo. It receives the forces/torques applied by the human hands at the two real handles as inputs, which drive the whole simulation of the system along with the virtual cobot controller in question. This module outputs the virtual handle motion as an input (reference) for a haptic rendering module, which uses a motion controller of the dual-arm system to control the real handles to track their virtual counterparts. The physical simulation module also outputs the motions of the virtual object and the virtual cobot, which are visualized by Unity and fed back to the operator through the VR headset, that is, visual rendering module. In this way, the haptic and visual feedback are combined to enhance the realism of the PHRC tasks to be emulated by the PREDICTOR.

### 2.3. Features and benefits

#### 2.3.1. Admittance-type haptic device

According to the control scheme of a haptic device, there are two broad classes of haptic devices: admittance-type and impedance-type devices. Admittance-type haptic devices sense the force/torque applied by the operator and constrain the operator's hand position to match the appropriate deflection or motion of a simulated object in a virtual world. In contrast, impedance-type devices sense the position of the operator, then applies a force/torque to the operator according to the computed behavior of the simulated object. In our PREDICTOR, the admittance type was chosen to implement the haptic rendering module for four reasons listed below:

- Admittance-type haptic devices can avoid developing an algorithm of collision detection between an avatar of the tool gripped by the operator and the objects in a virtual environment and an algorithm of force response for calculating the interactive force between them in order to drive the simulation (Salisbury et al., 2004). In our case, the virtual handle (avatar) is already attached to (in contact with) the virtual object. It is more convenient to use the force/torque sensed by a Force/Torque (F/T) sensor mounted between the handle and the end-effector of the real robot to drive the simulation instead of using the collision detection and force response algorithms to transform the motion input to the force input for the simulation.
- Since PREDICTOR is targeted at the safety and ergonomics research in PHRC, the real interactive force in an admittance-type haptic device is more desirable for human behavior study than the artificial interactive force calculated by the collision detection and force response algorithms in an impedance-type haptic device.
- A motion controller in the haptic rendering of an admittance-type device is in favor of the implementation of the kinematic constraint between the two real handles imposed by the rigid body constraint between their virtual counterparts attached to the same object.
- Impedance-type haptic devices require the real robots to be torque-controlled. However, more commercially available robots are position-controlled, e.g., Universal Robots. Selection of the admittance type can widen the uptake and applications of the PREDICTOR.

#### 2.3.2. Risk-controlled and flexible emulator

When a human coworker and a cobot co-manipulate one heavy and bulky object (e.g., a car engine), it is risky to validate the safety of the whole process through actual experiments since any accidents in the experiments can seriously hurt the human coworker, for instance, the abnormal behavior of the designed cobot controller can generate a large adverse force on the coworker and cause joint injuries or make the object fall down and hurt his/her legs or feet. Through the PREDICTOR, it is beneficial to analyze the operator reactive behavior in response to the cobot, evaluate the risk of the task in terms of various biomechanical quantities of the human body (e.g., muscle force and joint torque), and conveniently intervene in the process to respond to emergent and dangerous events (e.g., halt the system in time when the monitored interactive force exceeds a safe threshold value) without all the safety concerns in relation to the real experiments. Therefore, PREDICTOR is a risk-controlled test bench to enable the assessment of whether the proposed human-robot collaboration solutions are safe, ergonomic and deployable.

In addition, PREDICTOR is a general and flexible platform for emulating various types of PHRC tasks. To test a different task, what we just need is to deploy a different controller on a virtual cobot and/or use another model for a different co-manipulated object without actually fabricating it out. If the positions of the virtual handles on the co-manipulated object are changed, the dual-arm system can be easily reconfigured to position the real handles consistent with their virtual counterparts. In this sense, the dual-arm system is better than a single arm with a rigid link connecting the two real handles to the arm end-effector since a new link needs to be fabricated every time the virtual handles' positions are changed. Through the PREDICTOR, we can figure out the best setting (e.g., the best handle positions), optimize the task and the controller (and even the mechanical design of the cobot), and train the operator before real PHRC experiments, which are generally costly in terms of the fabrication cost and the preparation of the whole setup.

## 3. Implementation of software modules

### 3.1. Physical simulation module

The open-source robotics simulator, Gazebo, is used in the physical simulation module to simulate a PHRC system, in which a virtual cobot and a virtual object can be modeled in URDF format and connected in Gazebo in a way depending on the PHRC task to be emulated. One or two virtual handle(s) as the avatar(s) of the real handle(s) is/are attached to the virtual object. The force/torque applied on the real handle(s) by the operator is sent into Gazebo and applied on their virtual counterpart(s) to drive the simulation along with a cobot controller to be examined. Because of the nature of PHRC tasks, an impedance controller (Hogan, 1984) can be chosen for the controller of the virtual cobot.

The dynamic model of a robot manipulator is expressed:


(1) $$\begin{array}{l} M\left(q\right)\ddot{q}+C\left(q,\dot{q}\right)\dot{q}+g\left(q\right)=\tau -J^{T}F_{ext}, \end{array}$$

where ***M***(***q***), $C\left(q,\dot{q}\right)$ and ***g***(***q***) denote the inertia matrix, Coriolis and centrifugal matrix and gravity vector of the manipulator, respectively. ***τ*** is the vector of robot joint torques and ***F***_*ext*_ is the force/torque applied by the robot through its end-effector on the external environment. ***J*** indicates the robot Jacobian. The goal of a Cartesian impedance controller is to design an appropriate ***τ*** to alter the natural dynamics in Equation (1) to make the robot's end-effector exhibit a virtual mechanical impedance behavior (Khatib, 1987), which can be described by


(2) $$\begin{array}{l} \Lambda_{d}\ddot{\tilde{x}}+D_{d}\dot{\tilde{x}}+K_{d}\tilde{x}=F_{ext}, \end{array}$$

with **Λ**_*d*_, ***D***_*d*_, and ***K***_*d*_ being the desired mass, damping, and stiffness matrices, respectively. $\tilde{x}$ denotes the error of the end-effector position and orientation compared to a desired reference. The feedback of ***F***_*ext*_ can be avoided when **Λ**_*d*_ is set to be identical to the robot natural inertia **Λ** (Ott, 2008). ***K***_*d*_ is designed according to the emulated PHRC task while ***D***_*d*_ can be further designed based on the designed ***K***_*d*_ by using the *double diagonalization design* approach (Albu-Schaffer et al., 2003). This Cartesian impedance controller is used for the virtual cobot in Gazebo in the validation experiments in Section 4.

### 3.2. Haptic rendering module

**Figure 3:**
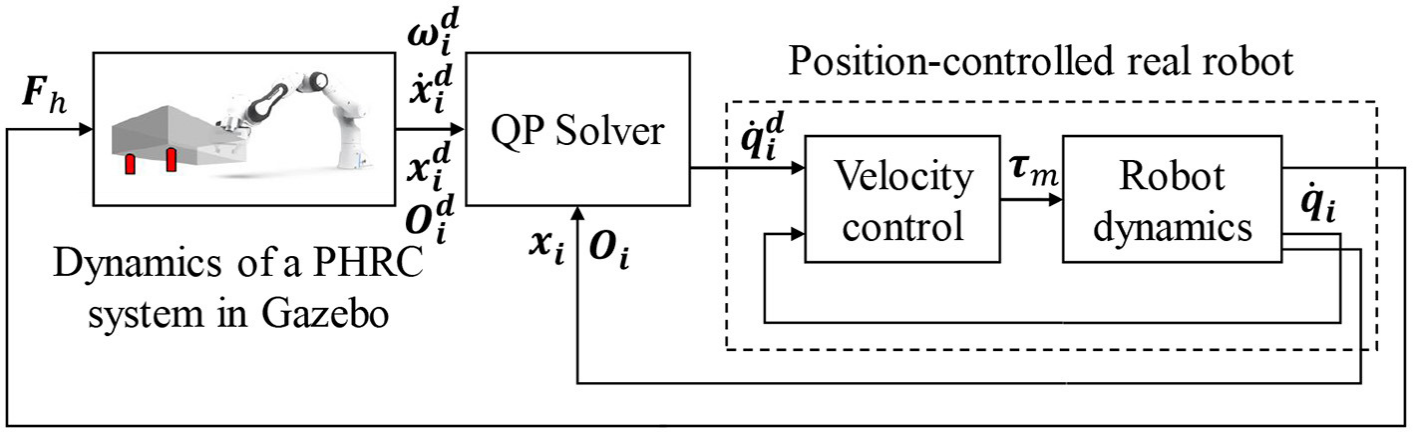
Implementation of the haptic rendering module based on an admittance-type control scheme.

Admittance-type control scheme is used to realize the haptic rendering module. As shown in Figure 3, the forces/torques, ***F***_*h*_, applied by the human operator on the two real handles are measured by F/T sensors, sent into Gazebo and applied on the same positions of the corresponding virtual handles. These forces/torques are monitored in real time and if they exceed predefined safe threshold values, the dual-arm robot system will be halted to guarantee the safety of the operator. The physical simulation of a virtual PHRC system in Gazebo will be driven by the transmitted forces/torques together with a controller of the virtual cobot. The motion states of the two virtual handles, i.e., the position and orientation ($x_{i}^{d}$, $O_{i}^{d},i=1\;\text{or}\;2$) and the linear and angular velocities (${\dot{x}}_{i}^{d}$, $\omega_{i}^{d}$), are measured and used as the targets the real handles try to track as much as possible. Traditionally, differential Inverse Kinematics (IK) techniques can be employed to solve for the corresponding desired joint velocities, ${\dot{q}}_{i}^{d}$. However, some constraints of real robots like the joint angle/velocity limits and singularities can make the performance of these IKs deteriorate. Quadratic Programming (QP) techniques are becoming more popular and robust to solve the IK problem by transforming the exact tracking problem into an optimization problem (i.e., minimizing the tracking error), especially when the numbers of the robot DoFs and constraints are large (Zhou et al., 2016). A QP solver is therefore used in our case to solve for ${\dot{q}}_{i}^{d}$, which will be used as the control command for our real dual-arm robot system in velocity control mode. The structure of this control scheme resembles that of a standard admittance control with an inner motion control loop and an outer admittance control loop. The difference is an impedance behavior is rendered at the end-effector of a robot through the outer loop in the admittance control while the dynamic behavior of a PHRC system exhibited at the end-effector(s) is rendered through the outer loop in our control scheme. The stability of the system can be ensured provided that the equivalent bandwidth of the inner control loop is larger than the equivalent bandwidth of the outer control loop (Siciliano et al., 2009).

A QP optimization problem has a general form as follows:


(3) $$\begin{array}{l} \underset{X}{\min}\;\frac{1}{2}X^{T}PX+c^{T}X, \end{array}$$

(4) $$\begin{array}{l} \text{s.t.}\;AX=b, \end{array}$$

(5) $$\begin{array}{l} GX\leq h, \end{array}$$

(6) $$\begin{array}{l} X_{\text{lb}}\leq X\leq X_{\text{ub}}, \end{array}$$

where X is the target variable to be optimized, and ***P***, ***c***, ***A***, ***b***, ***G***, ***h***, $X_{\text{lb}}$ and $X_{\text{ub}}$ are the parameters to form the problem-specific objective functions and constraints. The construction of these matrices and vectors in our QP optimization problem will be introduced in the following.

#### 3.2.1. Objective function

In this study, the joint velocities of the two robots are chosen and stacked as the unknown column vector variable, i.e., $X={\left({\left({\dot{q}}_{1}^{d}\right)}^{T},{\left({\dot{q}}_{2}^{d}\right)}^{T}\right)}^{T}\in {\mathbb{R}}^{2n}$ (assume each robot has *n* controlled joints.). In order to make the real handles to track the motion of their virtual counterparts as much as possible, the objective function is designed as:


(7) $$\begin{array}{l} \underset{X}{\min}\;\frac{1}{2}∥JX-v^{r}{∥}^{2}, \end{array}$$

where ∥·∥ is Euclidean norm, ***J*** is the Jacobian of the dual-arm robot system and ***v***^*r*^ is the reference velocities of the two real handles calculated by the *velocity-based control* (Nakanishi et al., 2008):


(8) $$\begin{array}{l} J=\left(\begin{array}{cc} J_{1} & O\\ O & J_{2} \end{array}\right)\in {\mathbb{R}}^{12\times 2n},\;v^{r}=\left(\begin{array}{c} v_{1}^{r}\\ v_{2}^{r} \end{array}\right)\in {\mathbb{R}}^{12}, \end{array}$$

(9) $$\begin{array}{l} v_{i}^{r}=\left(\begin{array}{c} {\dot{x}}_{i}^{d}+K_{p}\left(x_{i}^{d}-x_{i}\right)\\ \omega_{i}^{d}+K_{o}\left(-\eta_{i}^{d}\epsilon_{i}+\eta_{i}\epsilon_{i}^{d}-S\left(\epsilon_{i}^{d}\right)\epsilon_{i}\right) \end{array}\right)\in {\mathbb{R}}^{6}, \end{array}$$

where ***J***_1_ and ***J***_2_ are the geometric Jacobians of the two robots. ***x***_*i*_ denotes the position of the real handle *i*. {η_*i*_, ***ϵ***_*i*_} and $\left\{\eta_{i}^{d},\epsilon_{i}^{d}\right\}$ are the unit quaternions of the real and virtual handles of robot *i*, respectively. *K*_*p*_ and *K*_*o*_ are the translational and rotational coefficients. ***S***(·) is a skew-symmetric operator. The objective function in Equation (7) can be equivalently rewritten to the general QP form as in Equation (3) where ***P*** = ***J***^*T*^***J*** and ***c*** = −***J***^*T*^***v***^*r*^ with a constant term $\frac{1}{2}{v^{r}}^{T}v^{r}$ dropped out.

Manipulability optimization can also be included in the objective function to drive the robot system to avoid the singularities for better tracking performance and stability. A translational manipulability Jacobian, $J_{m}\in {\mathbb{R}}^{2n}$, which relates the change rate of manipulability of the dual-arm system to its joint velocities X, can be calculated by the robot Hessian tensors introduced in Haviland and Corke (2021). In this case, $c=-J^{T}v^{r}-\alpha J_{m}$ can be used with a scalar weight α for balancing the handle tracking and the manipulability optimization.

#### 3.2.2. Rigid body constraint between two handles

**Figure 4:**
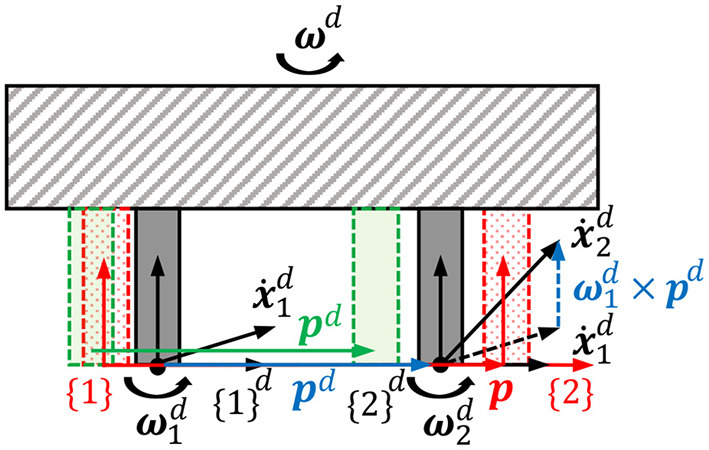
Rigid body constraint between the two real handles imposed by their virtual counterparts, which are attached to the same rigid body in the simulator.

By minimizing the objective function, the real handles try to track the motions of the virtual handles as much as possible, which means the tracking errors are allowed to occur in some cases where the tracking performance is sacrificed by optimizing the manipulability more or the robot system is close to its singular configuration. In those cases, the relative position and orientation between the two real handles still remain unchanged as a rigid body constraint imposed by their virtual counterparts attached to the same rigid body. This means that the proprioception of relative position between two hands is prioritized over the exteroception of exact positions of the handles for better realism perceived by users. For instance, the green handles are considered a better tracking compared to the red handles shown in Figure 4 because the distance between green handles are the same as the distance between the gray handles, which represent the virtual handles in the simulator. This rigid body constraint is implemented by the equality constraint (Equation 4) at the velocity level. Assume two reference frames, {1}^*d*^ and {2}^*d*^, are attached to the two virtual handles while {1} and {2} are attached to the two real handles shown in Figure 4. The angular velocities of {1}^*d*^ and {2}^*d*^, $\omega_{1}^{d}$ and $\omega_{2}^{d}$, are identical because angular velocity is an attribute of the rigid body and independent of the position of reference frame. The linear velocities of the origins of the two frames, ${\dot{x}}_{1}^{d}$ and ${\dot{x}}_{2}^{d}$, holds a relationship ${\dot{x}}_{2}^{d}={\dot{x}}_{1}^{d}+\omega_{1}^{d}\times p^{d}$ where symbol × denotes cross product and ***p***^*d*^ is a displacement vector from handle 1 to handle 2. So, the equality constraint between the velocities of {1} and {2} can be formulated as:


(10) $$\begin{array}{l} AX=b,\\ \;A=\left(\begin{array}{cc} J_{1l}+S{\left(p\right)}^{T}J_{1a}, & -J_{2l}\\ J_{1a}, & -J_{2a} \end{array}\right)\in {\mathbb{R}}^{6\times 2n},\\ \;b=\left(\begin{array}{c} -K_{p}^{\prime }\left(p^{d}-p\right)\\ -K_{o}^{\prime }\left(-\eta_{1}\epsilon_{2}+\eta_{2}\epsilon_{1}-S\left(\epsilon_{1}\right)\epsilon_{2}\right) \end{array}\right)\in {\mathbb{R}}^{6}, \end{array}$$

where $J_{il}\in {\mathbb{R}}^{3\times n}$ and $J_{ia}\in {\mathbb{R}}^{3\times n}$ are the upper and lower submatrices of ***J***_*i*_ which influence the linear and angular velocities of handle *i*, respectively, that is, $J_{i}={\left(J_{il}^{T}\;J_{ia}^{T}\right)}^{T}$. The equality constraint in Equation (10) tries to eliminate the relative translational and rotational tracking errors (***b***) between the two real handles to make them obey the rigid body motion law as much as possible. For example, the red handles in Figure 4 can be constrained to move to the green ones even though the absolute handle tracking is not perfect, which is handled by the objective function (Equation 7).

#### 3.2.3. Collision avoidance constraint

**Figure 5:**
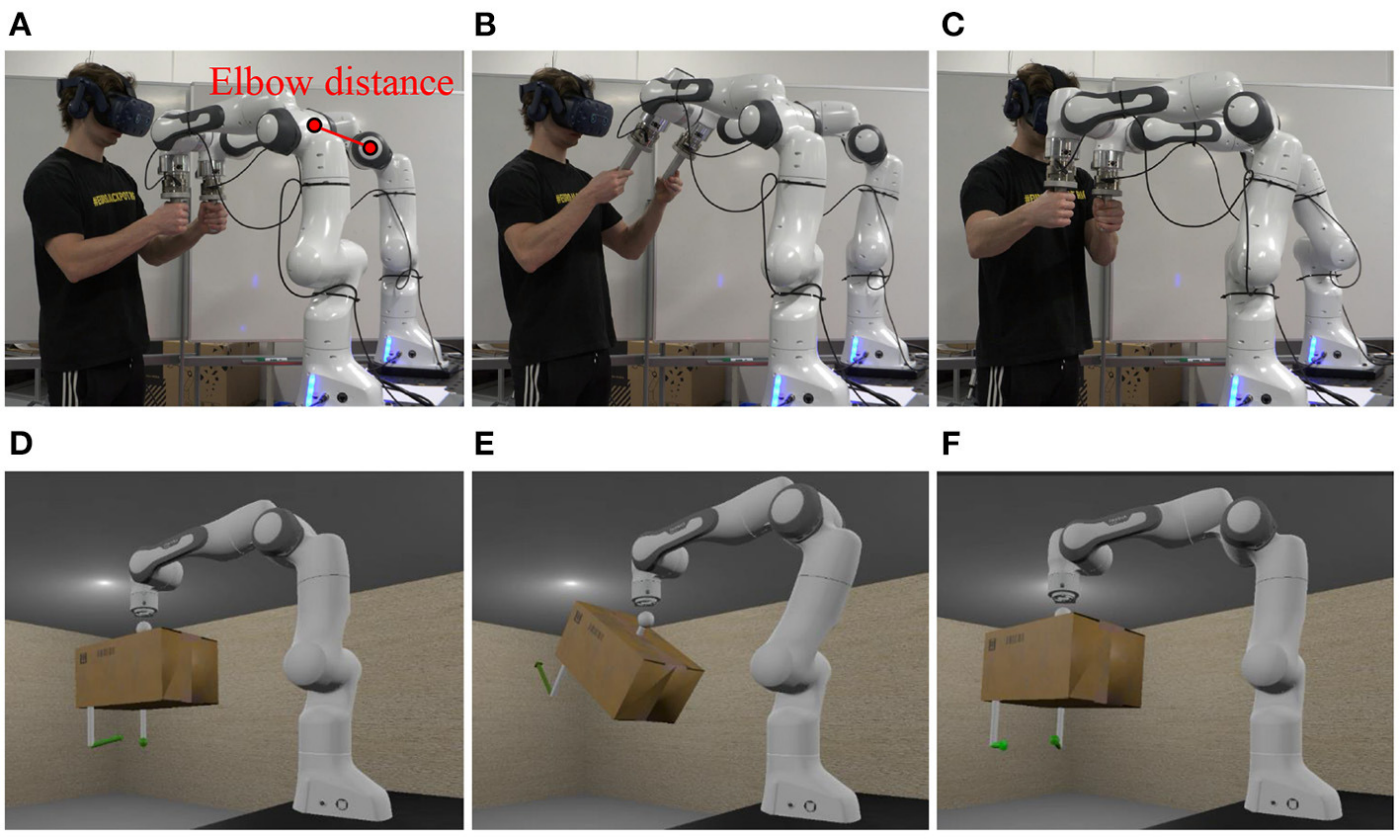
Snapshots of an experiment of scenario 1 at 5s **(A)**, 18s **(B)** and 28s **(C)**, and the corresponding snapshots of the VR scene in the visual rendering module at 5s **(D)**, 18s **(E)**, and 28s **(F)**.

The method named *velocity damper* (Faverjon and Tournassoud, 1987) is introduced for collision avoidance between two robot arms. Since the two bases and the two end-effectors are constrained apart from each other, respectively, only elbow collision avoidance is focused here. Elbow distance, *d*, is defined as the distance between the origins of two link frames located at the two elbow centers shown in Figure 5A. If the two elbows move closer to each other, the change rate of *d* is constrained as


(11) $$\begin{array}{l} \dot{d}\geq -\xi \frac{d-d_{s}}{d_{i}-d_{s}},\;\text{for }d<d_{i}, \end{array}$$

where ξ is the positive damping coefficient. Security distance, *d*_*s*_, is the minimum distance that *d* could be. This inequality implies that, when *d* is smaller than an influence distance, *d*_*i*_, the two elbows will try to decrease their approaching speed and prevent themselves being too close since *d* could never be smaller than *d*_*s*_.

Computing *ḋ* using the current configuration and the joint velocities of the dual-arm system, the *velocity damper* inequality becomes


(12) $$\begin{array}{l} n^{T}\left(J_{e1},-J_{e2}\right)X\geq -\xi \frac{d-d_{s}}{d_{i}-d_{s}},\;\text{for }d<d_{i}, \end{array}$$

where ***n*** ∈ ℝ^3^ is the unit directional vector from the center of elbow 2 to that of elbow 1. $J_{ei}\in {\mathbb{R}}^{3\times n}$ is the translational elbow Jacobian of robot *i*. Therefore, $G=-n^{T}\left(J_{e1},-J_{e2}\right)$ and $h=\xi \frac{d-d_{s}}{d_{i}-d_{s}}$ can be used to form the inequality constraint (Equation 5). Note that more pairs of links can be added into the collision avoidance constraint and more advanced collision avoidance techniques can be also incorporated (Fang et al., 2015).

#### 3.2.4. Joint angle/velocity limit constraint

Using the same *velocity damper* method, the lower and upper bounds of the *j*-th joint velocity in Equation (6) can be defined as follows,


(13) $$\begin{array}{l} f_{\text{min}}\left(q_{j}\right)\leq {\dot{q}}_{j}\leq f_{\text{max}}\left(q_{j}\right),\;\text{for }j=1,2,\cdots \;,2n, \end{array}$$

Where *f*_min_(*q*_*j*_) and *f*_max_(*q*_*j*_) are the functions of *q*_*j*_ as follows,


(14) $$\begin{array}{l} f_{\text{min}}\left(q_{j}\right)=\{\begin{array}{ll} -\xi_{q}\frac{\left(q_{j}-q_{j}^{-}\right)-q_{s}}{q_{i}-q_{s}} & \;\text{if }q_{j}-q_{j}^{-}\leq q_{i}\\ {\dot{q}}_{j}^{-} & \;\text{otherwise}, \end{array} \end{array}$$

(15) $$\begin{array}{l} f_{\text{max}}\left(q_{j}\right)=\{\begin{array}{ll} \xi_{q}\frac{\left(q_{j}^{+}-q_{j}\right)-q_{s}}{q_{i}-q_{s}} & \;\text{if }q_{j}^{+}-q_{j}\leq q_{i}\\ {\dot{q}}_{j}^{+} & \;\text{otherwise}, \end{array} \end{array}$$

where ξ_*q*_, *q*_*i*_ and *q*_*s*_ correspond to ξ, *d*_*i*_ and *d*_*s*_ in Equation (12), respectively. $q_{j}^{-}$ and $q_{j}^{+}$, ${\dot{q}}_{j}^{-}$ and ${\dot{q}}_{j}^{+}$ are the lower and upper physical limits of the *j*-th joint angle/velocity, respectively. Therefore, a total number of 2*n* bound constraints are introduced to form (Equation 6) for the QP problem of the dual-arm system in order to impose the joint angle/velocity limit constraints in a unified way.

Once all the objective function and constraints in Equations (3)–(6) are formulated, the complete QP optimization problem can be solved by state-of-the-art QP solvers efficiently (Vandenberghe, 2010; Ferreau et al., 2014). Note that single-arm emulator can be also realized by simply removing the rigid body constraint (Equation 10) and the collision avoidance constraint (Equation 12).

### 3.3. Visual rendering module

Visual rendering module is used to visualize what happens in the physical simulation module (in Gazebo) and provide the operator with visual feedback through a VR headset in order to help immerse the operator in the emulated tasks. Unity is chosen as the environment to develop the visual rendering module because it is a mature platform with good compatibility with various operating systems and VR headsets, and is widely used in many applications.

A robot model is needed to visualize a robotic system in Unity. As the URDF format is used in the physical simulation module to describe the robot and the object of a PHRC system, a URDF importer in Unity is therefore used to construct the system in Unity for consistency in modeling between the two modules. A system model is constructed by the URDF importer using *GameObjects* and *Articulation Bodies* from Unity. Inertial parameters of the links (*GameObjects*) in the model are circumvented manually after importing and the parameters of *Articulation Bodies* for simulating the dynamic behavior of an articulated system are disabled, because Unity is only used for visualization rather than physical simulation, which is already done in Gazebo. Likewise, default PD controller for driving each joint to a desired angle is replaced by simply setting the joint angle to the desired value received from the physical simulation module.

In order to receive the motion state of a PHRC system from the physical simulation module, a ROS-TCP-Connector plugin is used in Unity. The plugin enables communication between ROS and Unity *via* a TCP connection. The plugin allows Unity to receive data from the Gazebo simulation through a ROS node, which sends data whenever it is available in a specified topic it subscribes to. In Unity, the plugin creates a ROS subscriber, which calls a function to extract the joint angles of the PHRC system and update its motion state in Unity when a new message is received.

## 4. Experiments and results

### 4.1. Objective performance evaluation

**Figure 6:**
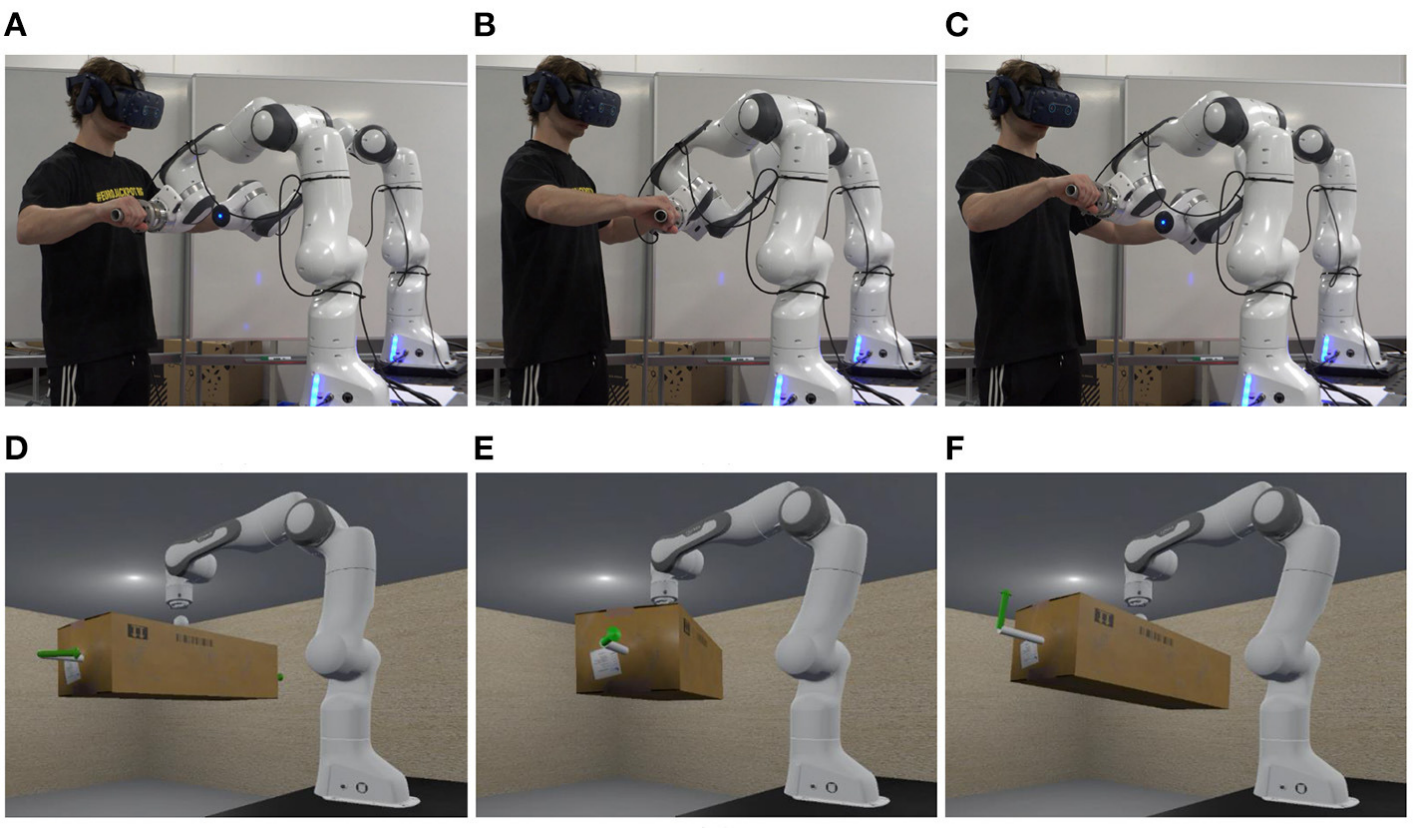
Snapshots of an experiment of scenario 2 at 5s **(A)**, 22s **(B)**, and 29s **(C)**, and the corresponding snapshots of the VR scene in the visual rendering module at 5s **(D)**, 22s **(E)**, and 29s **(F)**.

Two experiments were conducted to validate the effectiveness and evaluate the performance of the proposed PREDICTOR. An operator used PREDICTOR to emulate a human-robot co-transportation of a box in two different scenarios in order to show the flexibility of PREDICTOR. In scenario 1 shown in Figure 5, a 9 kg virtual box has a dimension of 0.26 meters wide (w), 0.2 meters high (h) and 0.38 meters long (l). Two virtual handles were attached to the bottom side of the box. In scenario 2 shown in Figure 6, another 18 kg box has a different dimension of 0.26(w) × 0.2(h) × 0.76(l) (meter). The two handles were attached to the left and right sides of the box. In both scenarios, the box was lifted by a 7-DoF virtual cobot with a Cartesian impedance controller in which the stiffness in the vertical direction is set to 500 N/m for assisting the operator in box lifting, while the stiffness in the other two horizontal directions is set to 5 N/m for allowing the operator to move the box around on a horizontal plane in the air. The reference set point is fixed. The cobot end-effector was connected through an unactuated spherical joint to a link, which is rigidly attached to the center of the top face of the box. This joint allows the operator to adjust the box orientation as well. Selected snapshots of the two experiments are shown in Figures 5, 6. Please see the accompanying video for the complete processes of the experiments. The presented research was approved by the Research Ethics Committee of the University of Southern Denmark.

The PREDICTOR is evaluated from the aspects of realism and safety.

#### 4.1.1. Objective realism (tracking performance)

**Figure 7:**
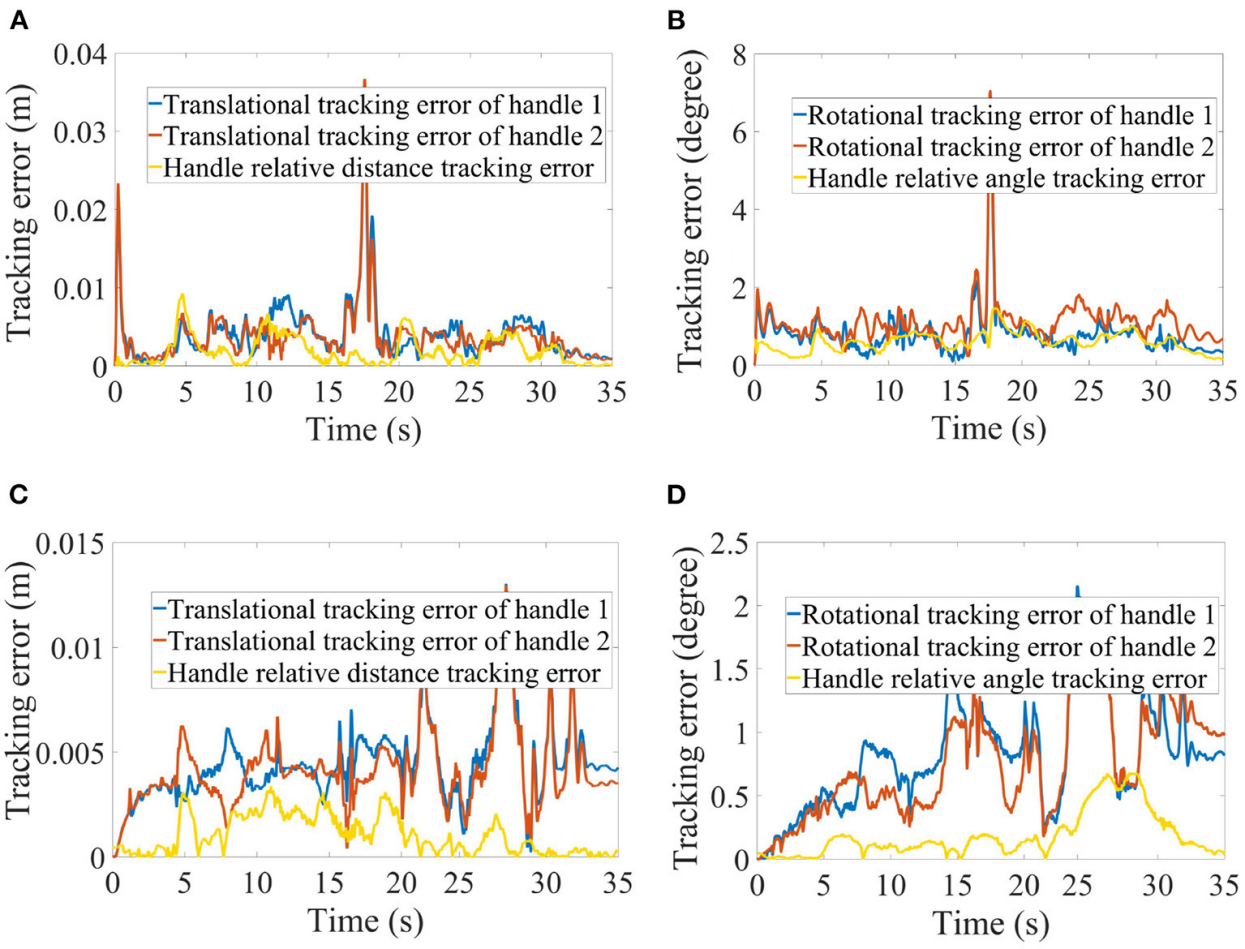
Translational tracking errors of the two handles and the relative distance between them in scenario 1 **(A)** and scenario 2 **(C)**, and rotational tracking errors of the two handles and the relative angle between them in scenario 1 **(B)** and scenario 2 **(D)**, respectively.

The objective evaluation of the realism was assessed by the absolute tracking errors of the two real handles compared against the motion of their virtual counterparts and the relative pose change between the two real handles. The translational tracking errors of the two handles were 4.1 ± 3.8 mm and 3.9 ± 3.8 mm in scenario 1, and 4.4 ± 1.9 mm and 4.1 ± 1.9 mm in scenario 2; the rotational tracking errors were 0.8 ± 0.6 degree and 1.1 ± 0.6 degree in scenario 1, and 0.8 ± 0.4 degree and 0.7 ± 0.4 degree in scenario 2; the handle relative distance and angle tracking errors were 1.9 ± 1.8 mm and 0.6 ± 0.3 degree in scenario 1, and 1.0 ± 0.9 mm and 0.2 ± 0.2 degree in scenario 2. All the error trajectories are shown in Figure 7. These results are consistent with our design in the QP formulation which tolerates the tracking errors (which are minimized rather than eliminated) and prioritizes the proprioception of the relative poses of two hands through haptic feedback (rigid body constraint in Equation 10) over the exteroception of the exact poses of two handles through visual feedback [objective function in Equation (7)]. This is manifested clearly in Figures 7A, B at 18-*th* second when the operator released the handles (Figure 5B). The absolute tracking was relatively poor (the spike) but the two handles' relative poses remained sufficiently good. *K*_*p*_ and *K*_*o*_ in Equation (9) were 2 and 0.4, and $K_{p}^{\prime }$ and $K_{o}^{\prime }$ in Equation (10) were 1.0 and 0.9 in both scenarios.

#### 4.1.2. Safety

**Figure 8:**
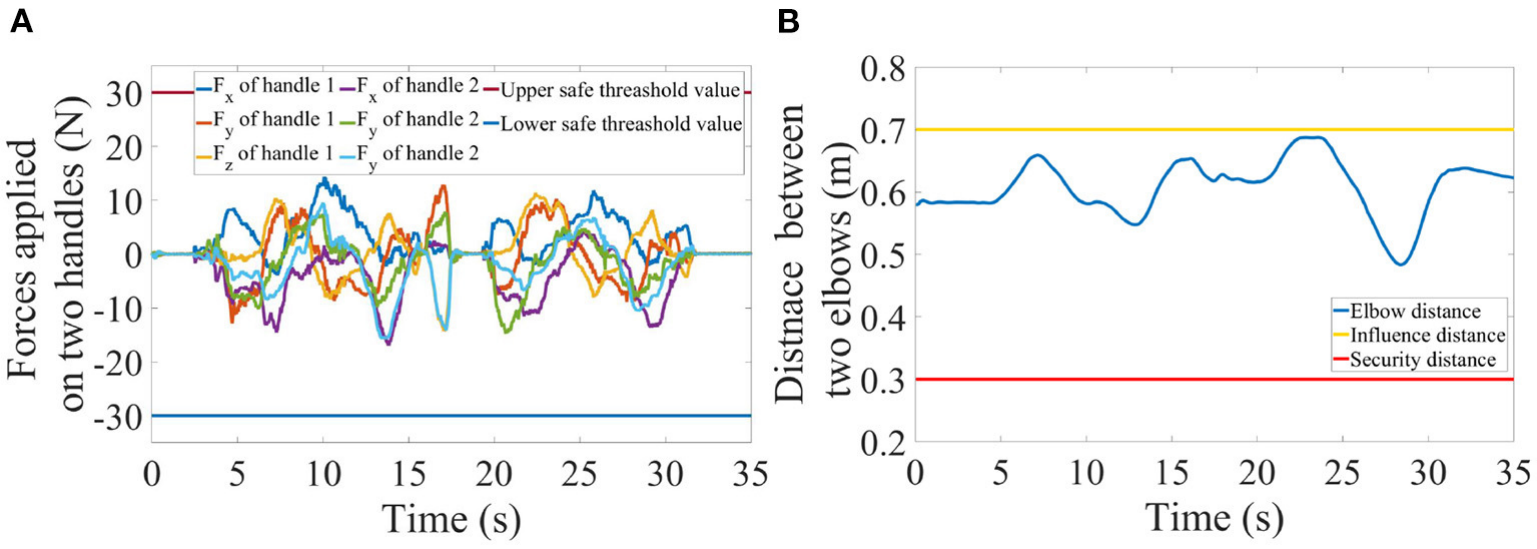
Profiles of the forces applied on the two handles **(A)** and the distance trajectory between the two elbows **(B)** in scenario 1.

The emulator safety was evaluated by the interactive forces/torques measured between hands and handles, and the distance between the two elbows of the dual-arm system indicated in Figure 5A. The former is a safety criterion for the human operator while the latter is a safety criterion for the system itself. As shown in Figures 5, 6, the measured forces were visualized by two green arrows on the virtual handles in the VR scenes, the color would change to orange and red to warn the operator of the potential risk if the force magnitude increases. The safe force and torque threshold values were set to 30N and 5Nm. *d*_*i*_ and *d*_*s*_ were set to 0.7 and 0.3 meters in Equation (12). The safety of the PREDICTOR was exemplified with the results of scenario 1 shown in Figure 8.

**Figure 9:**
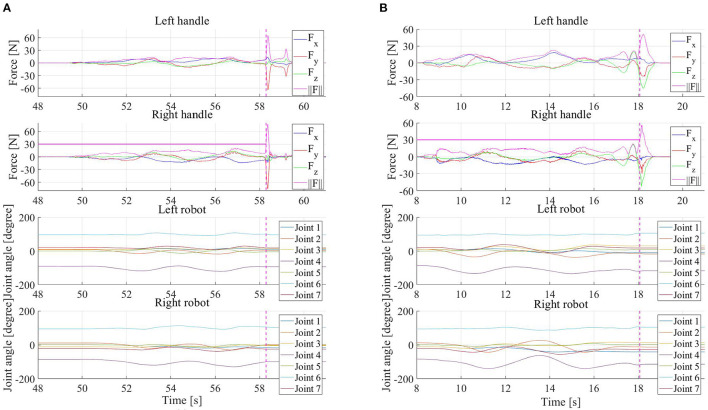
Force profiles of two handles applied by operator hands and joint trajectories of the two robots of PREDICTOR when the operator suddenly applied a force larger than a force limit of 30N **(A)**, and those when the Cartesian impedance controller of the virtual robot in the simulator was underdamped in vertical direction **(B)**. The dashed vertical pink lines indicate when the system was halted because the handle force magnitude reached the limit and the horizontal pink lines show which handle triggered the safety mechanism to halt the system in the two experiments.

In addition, two more experiments in scenario 1 were conducted to validate the developed emulator is a safe environment. In the first experiment, the operator intentionally applied a force larger than the force threshold of 30N suddenly, the system stopped immediately. In the second experiment, the Cartesian impedance controller of the virtual collaborative robot was underdamped in the vertical direction so the robot could not lift the box stably, and the system tended to oscillate in the vertical direction. During the operation of the system, the applied force by the operator easily exceeded the force threshold due to the oscillatory behavior, which triggered the action of halting the system successfully. The handle force profiles and the joint trajectories are shown in Figure 9 and the details of the two experiments can be found in the accompanying video.

### 4.2. Subjective user study

**Figure 10:**
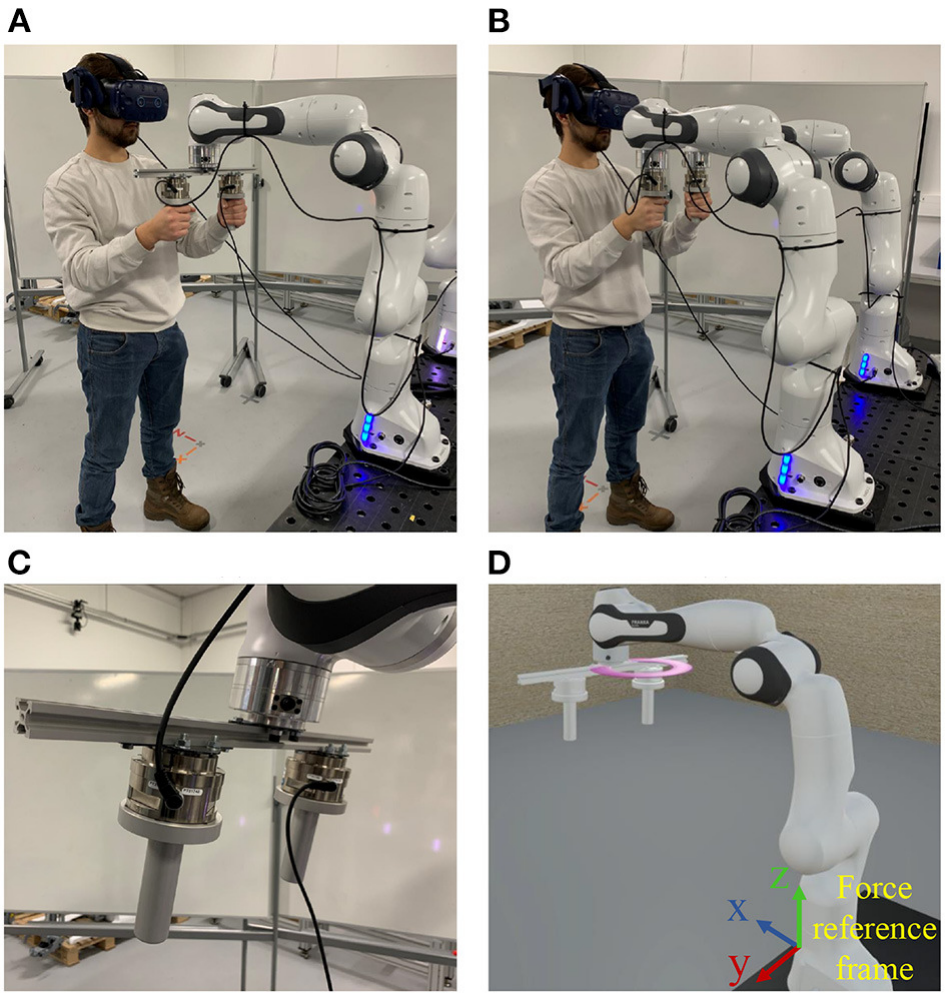
The real experiment setup **(A)** and the emulated experiment setup **(B)** of a physical human-robot co-manipulation of an aluminum bar **(C)** in the user study. Subjects operated the two setups to make the end-effector of a virtual robot in the same VR scene **(D)** move along the purple circle for four cycles at as constant speed as possible in the comparative experiments.

In addition to the objective evaluation of PREDICTOR as above, the subjective evaluation was performed with a user study. The goal of the user study was to evaluate subjective aspects of the proposed emulator compared to the real experiment/scenario it tries to emulate. As shown in the real experiment setup of a PHRC task in Figure 10A, a real aluminum bar in Figure 10C was used as a co-manipulated object, which was rigidly connected to the end-effector of a single Panda robot and to which two F/T sensors were connected. The bar together with the sensors and the connected handles weighed 2.1 kg in total. For the corresponding emulated experiment setup shown in Figure 10B, thanks to the flexibility offered by PREDICTOR, the only major change compared to the experiments in Section 4.1 was a modified URDF file of the co-manipulated object, which was used both in the physical simulation and the visual rendering modules. Apart from that, the three modules of PREDICTOR remained unchanged, which showed the desirable flexibility, i.e., fast reconfiguration of the system for emulating different scenarios. For a fair comparison between experiments using the two setups, subjects were asked to wear the same VR headset to see exactly the same VR scene shown in Figure 10D in both setups. In the real setup, the configuration of the real Panda was fed back to the visual rendering module instead of the configuration of the virtual Panda in Gazebo in the emulated setup. A purple circle was located on a horizontal plane in the VR scene, and the same task the subjects were asked to do in both setups is to operate both systems to make the end-effector of the virtual Panda move along the purple circle for four cycles at as constant speed as possible.

13 male and 4 female subjects participated in the user study with age of 23.12 ± 2.63 years. None of them had any experience with the two systems before. The subjects were briefed about the purpose of the experiments and the experiment procedure with an instruction sheet, and gave an informed consent to the participation. Each of the subjects operated the two systems to perform the same human-robot collaboration task introduced before. After the experiments, they reported the degree of agreement with several statements about the emulator performance from different aspects compared to the real system it emulates in a Likert-type questionnaire. The statements are listed below:

- *S1: The physical emulator is easy-to-use for the human-robot collaboration task*.
- *S2: The realism level of visual rendering of the emulation of the human-robot collaboration task is very good compared to the real task*.
- *S3: The realism level of haptic rendering/feedback of the emulation of the human-robot collaboration task is very good compared to the real task*.
- *S4: The overall realism level of the emulation of the human-robot collaboration task is very good compared to the real task*.
- *S5: The emulated human-robot collaboration task is as responsive as the real task*.
- *S6: I feel safe to use the physical emulator in the human-robot collaboration task*.
- *S7: I feel comfortable with the physical emulator in the human-robot collaboration task*.
- *S8: I am satisfied with the overall performance of the physical emulator*.

**Figure 11:**
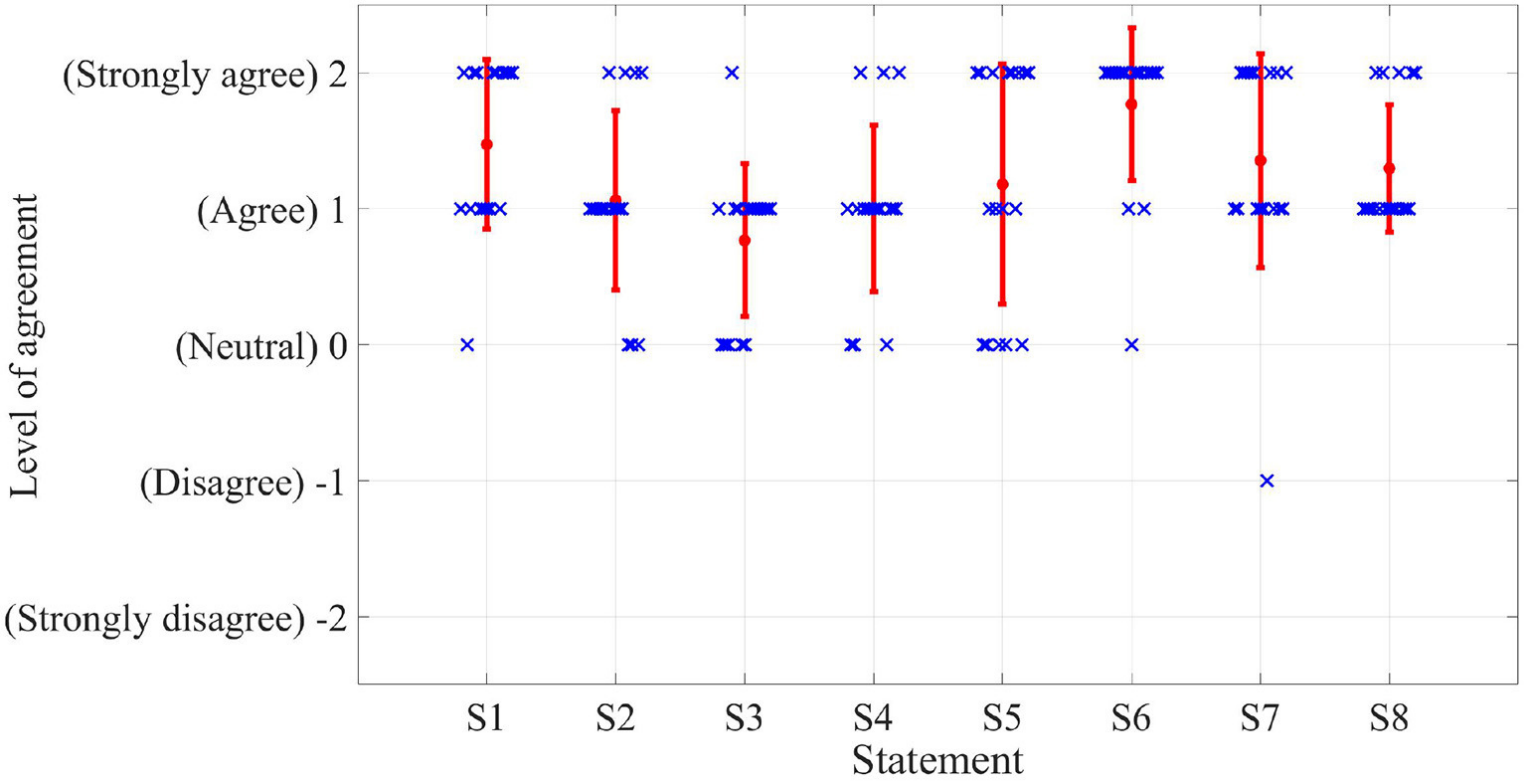
Detailed results of the user study for the subjective evaluation of PREDICTOR. The eight statements are listed on the x-axis while the level of agreement in terms of score is shown on the y-axis. The red dots and bars represent the means and standard deviations while blue crosses indicate the specific scores for different statements from all the subjects.

There were five levels of agreement (score is in the brackets): Strongly agree (2), Agree (1), Neutral (0), Disagree (–1), Strongly disagree (–2). The means and standard deviations of the degree of agreement with the eight statements are listed in the Table 1, and the detailed agreement levels of all the subjects are shown in Figure 11.

**Table 1:** Results of subjective evaluation of PREDICTOR from different aspects.

**Aspects**	**Statements**	**Agreement**
Usability	S1	1.47 ± 0.62
	S2	1.06 ± 0.66
	S3	0.76 ± 0.56
Subjective realism	S4	1.00 ± 0.61
Responsiveness	S5	1.18 ± 0.88
Perceived safety	S6	1.76 ± 0.56
Comfort	S7	1.35 ± 0.79
Overall performance	S8	1.29 ± 0.47

The values represent the means and standard deviations of the degree of agreement to the statements *S1*–*S8*. Positive value indicates agreement, while negative one indicates disagreement with the statement.

#### 4.2.1. Subjective realism

On average, the subjects agreed that PREDICTOR can provide very good realism level of the emulation compared to the real experiment task (1.00 ± 0.61), and the realism level of the visual rendering (1.06 ± 0.66) is slightly better than that of the haptic rendering (0.76 ± 0.56). For the statements *S2*–*S4*, there was no degree of agreement below Neutral (0). In the subjects' comments, some of them reflected that larger resistance was experienced in the real task system while the emulated task system was easier to move around, which contributed to the different haptic feeling when using the two systems. The reason for this different feeling is that the estimate of the joint damping in the model of the virtual Panda robot in Gazebo (joint damping of 1Nms was used) was probably less than that in the real Panda robot. That is why subjects may find harder to move the real robot. This can be improved by more accurate joint dynamic model through parameter identification methods, which will be part of our future work.

#### 4.2.2. Responsiveness

The responsiveness of the system was described by the time delay between the haptic rendering and the visual rendering modules. This time delay was mainly caused by the calculation time of the QP solver. This time was 10 ± 1 ms in the emulated scenario. According to the result of the agreement with the statement *S5* in the user study, the subjects did not perceive a noticeable time delay in the emulated experiments (1.18 ± 0.88). The QP formulation was implemented in Python. Better responsiveness can be expected if it is implemented in C++.

#### 4.2.3. The other aspects

In general, the subjects agreed that the developed emulator felt safe (1.76 ± 0.56), comfortable (1.35 ± 0.79) and easy to use (1.47 ± 0.62). Only one subject reported that the VR rendering made him feel uncomfortable. The mean and standard deviation of the level of agreement with the overall performance statement (*S8*) were 1.29 and 0.47, respectively. It is worth noting that there was no level of agreement below Agree (1) for the overall performance statement. All these results about different aspects manifest that the developed PREDICTOR has good performance and meets our expectations very well.

**Figure 12:**
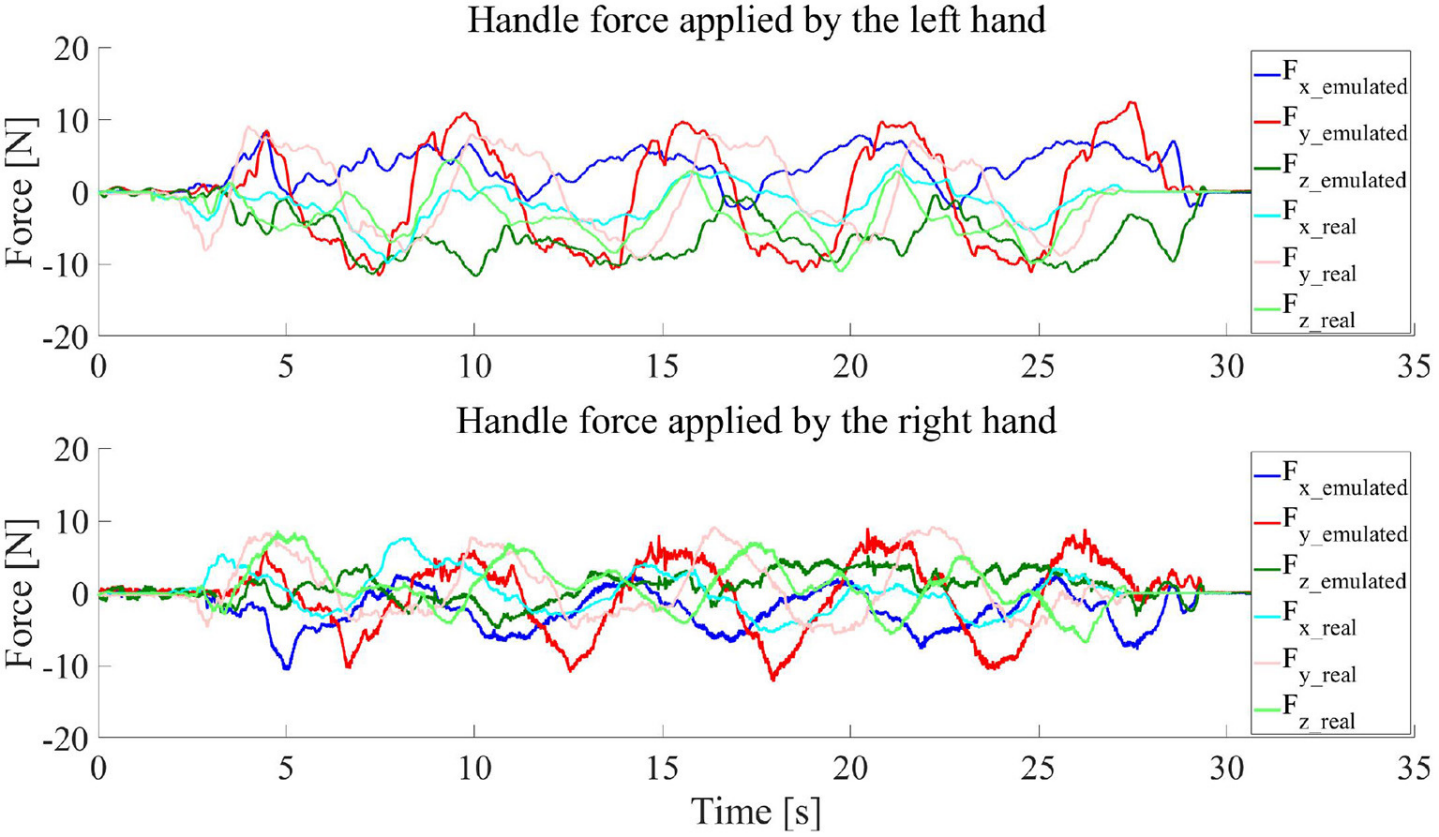
Handle forces applied by the operator hands when the operator used the real and emulated systems to move the end-effector of the virtual robot seen in the VR scene along a circular trajectory for four cycles.

The handle force profiles of one of the subjects using the two systems are given in Figure 12. As shown in the figure, the profiles of the forces applied by the two hands in x- and y-axes have very similar oscillatory patterns, i.e., similar start time, period and peak of each cycle, with the two systems. The difference in the force profile pattern in z-axis (see the force reference frame in Figure 10D) was ascribed to the freedom of applying arbitrary force in the vertical direction by the operator in this task, i.e., the robot helped lift the aluminum bar. In addition, our current limitation in accuracy for the joint damping estimation could also probably cause that the force pattern differed a bit between the two systems. It is worth noting that it is generally hard for an operator to operate the two systems at the same speed and in the same way even with a visualized reference trajectory. According to the observed similarity in the force pattern, it is believed that the developed PREDICTOR can be used to stimulate similar human interactive behavior in the emulated human-robot collaboration. More details of the example comparative experiments shown here can be found in the accompanying video.

### 4.3. Use of PREDICTOR for safety and ergonomics evaluation and training

Since the interactive process of a PHRC task can be emulated by PREDICTOR in a risk-controlled manner, detailed biomechanical (and cognitive) assessment during the process can be assessed by using different sensors. For instance, by attaching motion markers and EMG sensors to the human body doing the emulated tasks, the measured marker positions and muscle activity levels together with the measured force applied by the human hands can be used as inputs for a human musculoskeletal model to estimate biomechanical quantities of the operator during the emulations, e.g., joint trajectories (Fang et al., 2018), Cartesian and joint stiffnesses (Fang et al., 2017; Ajoudani et al., 2018a), joint torques (Peternel et al., 2021) and muscle forces (Peternel et al., 2019). These estimates can be further used to evaluate the safety and ergonomics of the emulated PHRC task. For instance, joint torques and muscle forces can be used to evaluate the safety (Kim et al., 2017) and human arm fatigue (ergonomics) (Peternel et al., 2019), respectively. The evaluation information can be then used to guide and improve the task and controller designs (including where to grasp the object, i.e., the handle positions or grasping points) to optimize the safety and ergonomics of the developed PHRC system before actual experiments.

In addition, as the environment the operator can see is a structured VR scene, useful operation instructions and individual task performance can be visualized in real time in the VR scene for novice users in order to improve their training experience and accelerate the training process. However, this paper focuses on the development and evaluation of the PREDICTOR, these in-depth evaluation and training studies based on the use of the PREDICTOR are out of the scope of this work, but will be definitely our future work.

## 5. Conclusion

In this paper, a physical emulator, PREDICTOR, was created to serve as a risk-controlled and flexible platform to emulate PHRC tasks for enabling the safety and ergonomics evaluation of PHRC solutions. PREDICTOR is comprised of a dual-arm cobot system and a VR headset. The physical emulation was implemented by physical simulation, haptic rendering and visual rendering modules. Gazebo was used to implement the PHRC simulation driven by the force/torque applied by a human operator. An admittance-type control scheme with a QP solver were employed to control the dual-arm system to act as an integrated haptic device and render the dynamic behavior simulated in Gazebo. Unity was used to visually feed the simulated motion back to the operator through the VR headset. Extensive experiments in different scenarios and a user study for the comparison between a real PHRC task and its emulated task through the PREDICTOR were performed to validate the efficacy and performance of PREDICTOR. The PREDICTOR opens the possibilities of conducting safety and ergonomics evaluation of designed potentially risky PHRC tasks in a safe environment emulated by the PREDICTOR, and also can be used to provide training service for new users before skillful operations in the actual tasks. It is expected to be used as a new tool and platform to facilitate and accelerate the relevant safety and ergonomics research in PHRC.

## Data availability statement

The raw data supporting the conclusions of this article will be made available by the authors, without undue reservation.

## Ethics statement

The studies involving human participants were reviewed and approved by Research Ethics Committee at University of Southern Denmark. The participants provided their written informed consent to participate in this study.

## Author contributions

CF and FC developed the concept and methods. CES, DTS, and CNPH contributed to programming, experiments, and data analysis. CF and CES wrote the first draft of the paper. FC, DTS, and CNPH revised the paper. All authors read and approved the submitted version.
